# How timely closure can reduce outbreak duration: gastroenteritis in care homes in North West England, 2012–2016

**DOI:** 10.1186/s12889-018-5413-x

**Published:** 2018-04-12

**Authors:** Thomas Inns, Alex Keenan, Rita Huyton, John Harris, Miren Iturriza-Gomara, Sarah J. O’Brien, Roberto Vivancos

**Affiliations:** 1 Field Epidemiology Service, Public Health England, Liverpool, UK; 2 Institute of Psychology, Health and Society, University of Liverpool, Liverpool, UK; 3 NIHR Health Protection Research Unit in Gastrointestinal Infections, University of Liverpool, Liverpool, UK; 4 Cheshire and Merseyside Health Protection Team, North West Centre, Public Health England, Liverpool, UK; 5 Division of Population Health, Health Services Research and Primary Care, School of Health Sciences, University of Manchester, Manchester, UK; 6 Institute of Infection and Global Health, University of Liverpool, Liverpool, UK; 7 NIHR Health Protection Research Unit in Emerging & Zoonotic Infections, University of Liverpool, Liverpool, UK

**Keywords:** Gastroenteritis, Gastrointestinal viruses, Noroviruses, Outbreaks, Surveillance, Infection control

## Abstract

**Background:**

Data on outbreaks of infectious gastroenteritis in care homes have been collected using an internet-based surveillance system in North West England since 2012. We analysed the burden and characteristics of care home outbreaks to inform future public health decision-making.

**Methods:**

We described characteristics of care homes and summary measures of the outbreaks such as attack rate, duration and pathogen identified. The primary analysis outcome was duration of closure following an outbreak. We used negative binomial regression to estimate Incidence Rate Ratios (IRR) and confidence intervals (CI) for each explanatory variable.

**Results:**

We recorded 795 outbreaks from 379 care homes (37.1 outbreaks per 100 care homes per year). In total 11,568 cases, 75 hospitalisations and 29 deaths were reported. Closure within three days of the first case (IRR = 0.442, 95%CI 0.366–0.534) was significantly associated with reduced duration of closure. The total size of the home (IRR = 1.426, 95%CI = 1.275–1.595) and the total attack rate (IRR = 1.434, 95%CI = 1.257–1.595) were significantly associated with increased duration of closure.

**Conclusions:**

Care homes that closed promptly had outbreaks of shorter duration. Care home providers, and those advising them on infection control, should aim to close homes quickly to prevent lengthy disruption to services.

## Background

Infectious gastroenteritis is a common cause of illness in care homes, which provide an environment well suited for the spread of infectious disease [1]. In a systematic review of published surveillance, the mean global incidence of infectious gastroenteritis in care home residents was estimated to be 0.40 (95% confidence interval 0.27–0.56) episodes per 1000 bed-days [2]. Norovirus is the most common cause of gastroenteritis outbreaks in care homes [3] and is associated with excess mortality in the elderly [4–6]. The majority of norovirus infections are transmitted person-to-person [7]. It is difficult to prevent transmission of norovirus because of its low infectious dose, lack of long term immunity to reinfection, and the fact that infected people can shed norovirus asymptomatically at high levels for at least 3 weeks [8].

Few surveillance systems capture the incidence of gastroenteritis in care homes [2]. Most contemporary surveillance data for infectious gastroenteritis outbreaks in care homes come from France or Australia. There is a national surveillance system in France which has published data from a two year period [9]. Care homes in one region of France are part of an enhanced surveillance system, which has been studied to better understand the aetiology and burden of infectious gastroenteritis outbreaks in that population [10, 11]. Similar surveillance is undertaken in Australia at national level, detailed epidemiological descriptions of outbreaks in this population are available [12].

In England, the Care Quality Commission (CQC) requires that care homes report significant outbreaks of infectious gastroenteritis to Public Health England (PHE) [13]. Information on general outbreaks of infectious gastroenteritis has been collected since 1992 [14]. However there is no dedicated national surveillance system for care home outbreaks.

In December 2012 a secure internet-based surveillance system was established in Cheshire & Merseyside in the North West of England to collect reports of care home outbreaks with agreement from the Cheshire & Merseyside Health Protection Team (CMHPT) and the relevant Infection Prevention and Control Teams. We analysed these surveillance data to provide insight into the burden and characteristics of care home gastroenteritis outbreaks and to inform future public health action.

## Methods

### Setting

This study took place in Cheshire & Merseyside, North West England. The area comprises nine Local Authorities and a total population of just under 2.5 million; 19% of the population are aged 65 and over [15]. The region had 535 care homes registered with the CQC as of 08 December 2016 [16]. Care homes in England must register with the CQC in accordance with Schedule 1 of The Health and Social Care Act 2008 (Regulated Activities) Regulations 2014.

### Definitions

A care home was defined as a residential long-term care facility, with or without nursing care. An outbreak of gastroenteritis was defined as two or more individuals with diarrhoea and/or vomiting within a care home, where symptoms were of a suspected infectious nature (not associated with prescribed drugs or treatments and not associated with an underlying medical condition or illness). Outbreaks of a bacterial aetiology or suspected food poisoning were excluded.

### Surveillance system

The local Community Infection Prevention & Control Team (CIPCT) is informed by the care home of an outbreak; the CIPCT team records this information using an internet-based questionnaire with the database stored on a secure server. The questionnaire collects information on the characteristics of the care home, details of the cases in residents and staff, and any microbiological testing. The database is accessed by CMHPT who produce routine surveillance reports for local public health stakeholders. The reports are followed up with the CIPCT teams to confirm details before final publication. Stool samples were tested at local laboratories; bacteriology using routine culture, parasitology using Enzyme-linked Immunosorbent Assays (EIA), *Clostridium difficile* using two stage testing as per national guidance which includes EIA [17], and virology using Polymerase Chain Reaction (PCR) tests.

### Analysis

This analysis includes outbreaks between 01 December 2012 and 31 November 2016, a four year period. We matched study surveillance data to CQC care home registration data extracted on 08 December 2016 [16]; data were matched using care home postcode and name. CQC data contains a four-stage rating of the care home based on inspection reports. This dataset covers currently registered care homes and has been updated monthly at a national level since September 2015.

We described characteristics of care homes reported to the surveillance system and summary measures of the outbreaks. We calculated attack rates using the total number of cases reported divided by the total number of residents and staff at the care home. The number of days that the care home was closed to new admissions or visitors was used as the outcome measure in multivariable analysis. We examined the association between this outcome and the following variables; total number of persons at the home, total attack rate, winter season, CQC overall rating, presence of residents with dementia, ratio of residents to staff and days between first case and closure. We categorised total number of persons at the home into quartiles, and total attack rate into three groups (under 20%, 20 to 39.9% and 40% and over). We used negative binomial regression to estimate Incidence Rate Ratios (IRR) and confidence intervals (CI) for each variable, using random-effects to account for clustering due to multiple outbreaks from the same care home. Descriptive analysis was conducted using R [18] and regression analysis was undertaken using Stata 13.1 (StataCorp, College Station, Texas).

## Results

**Fig. 1:**
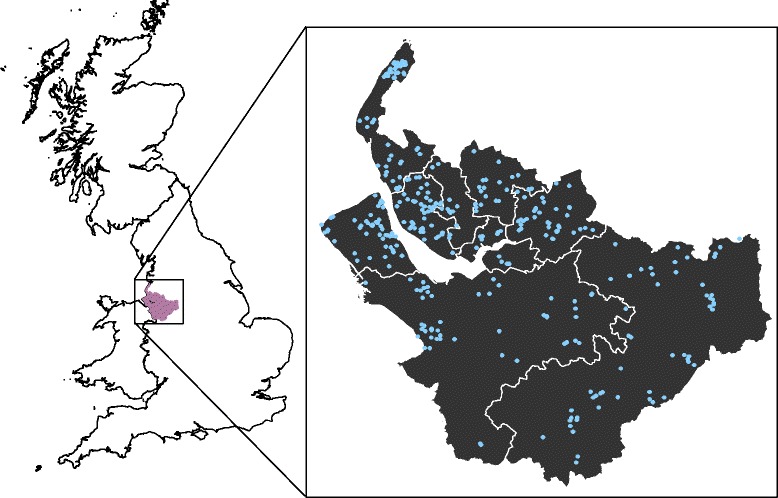
Geographical area covered by surveillance and location of care homes reported outbreaks (*n* = 379), November 2012–December 2016

From 1 December 2012 to 31 November 2016, 795 outbreaks were recorded from 379 care homes. Over the four year study period this equates to a rate of 37.1 outbreaks per 100 care homes per year. More than one outbreak was reported by 47.7% (181/379) of care homes; the highest number of outbreaks reported by one home in this period was 19. The number and rate of outbreaks in all nine Local Authorities in the Cheshire & Merseyside area are shown in Table 1. The greatest number of outbreaks was reported in Local Authority E (160), but the greatest rate of outbreaks was reported from Local Authority H (60 outbreaks per 100 care homes per year). The geographical distribution of care home outbreaks is shown in Fig. 1.

**Table 1:** Number and rate of outbreaks by local authority, November 2012–December 2016

Local authority	Number of outbreaks	Registered care homes	Rate per 100 care homes per year
A	131	79	41.46
B	126	75	42.00
C	31	23	33.70
D	20	27	18.52
E	160	74	54.05
F	115	116	24.78
G	36	34	26.47
H	84	35	60.00
I	92	111	20.72

The median number of residents was 34 (range 7–112) and the median number of staff was 36 (range 7–160). The surveillance data could not be linked to CQC data for 113 (29.8%) care homes. Of the 266 care homes with CQC information, all were in operation in December 2016 and the following overall ratings were given; inadequate (11), requires improvement (103), good (150) and outstanding (2).

**Fig. 2:**
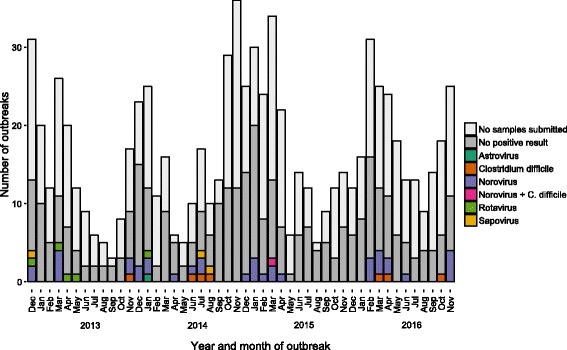
Number of care home outbreaks, showing those with samples submitted and the result, November 2012–December 2016

Reported outbreaks exhibited a winter seasonal distribution; outbreaks were most commonly reported in November (92), December (91), January (91), February (78) and March (101). The distribution of outbreaks by month is shown in Fig. 2. This figure also shows the outbreaks for which a sample was submitted for microbiological analysis; at least 1 sample was submitted for 356 (44.7%) outbreaks. The following pathogens were detected; norovirus (37), *Clostridium difficile* (7), rotavirus (5), sapovirus (3), astrovirus (1), mixed pathogens (norovirus and *C. difficile*) (1). Although included in the testing program, no *E. coli* O157 or *Salmonella* was detected. No positive result was recorded for 302 (85%) of outbreaks where a sample was submitted.

In total 11,568 cases (8539 residents and 3029 staff) were reported as part of the 795 outbreaks. Of these, 75 cases were hospitalised (69 residents and 6 staff) and 29 residents were reported to have died. The median attack rate was 6% in staff and 30% in residents The median attack rate was 17.6% of all persons per home (range 1.2% - 100%); the attack rate was under 20% in 448 outbreaks, between 20% and 39.9% in 286 outbreaks and over 40% in 64 outbreaks. The median ratio of residents to staff was 0.89 (range 0.17 to 5.86).

**Fig. 3:**
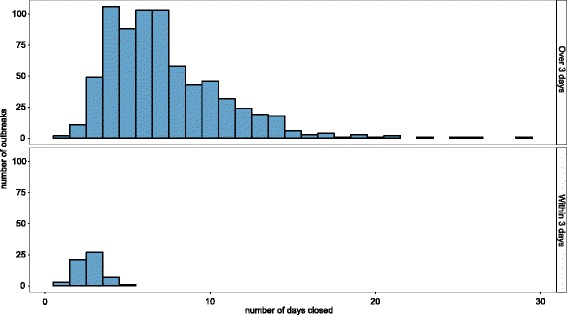
Duration of closure for each outbreak recorded in the surveillance system (*n* = 795), stratified by whether the closure was within 3 days of the first case, November 2012–December 2016

Care homes were closed for a median of 6 days during an outbreak (range 1–29 days). The distribution of length of closure is shown in Fig. 3. The median time between the first case and the care home closure was 8 days (range 2–29 days); 59 homes closed within 3 days of the first case, compared to 657 which closed more than 3 days after the first case. The homes which closed within 3 days of the first case remained closed for a median of 3 days (range 1–5) compared to a median of 7 days (range 1–29) for homes that closed more than 3 days after the first case.

Results of the negative binomial regression analysis are shown in Table 2. In univariable analysis, the duration of closure was significantly associated with: the total size of the home (IRR = 1.374, 95% CI = 1.244–1.517 in the largest quartile); total attack rate (IRR = 1.370, 95% CI = 1.213–1.547 for outbreaks with an attack rate over 40%); presence of residents with dementia (IRR = 1.106, 95% CI = 1.033–1.184); ratio of residents to staff (IRR = 0.914, 95% CI = 0.846–0.986); and closure of the home within 3 days of the first case (IRR = 0.371, 95% 0.311–0.441).

**Table 2:** Negative binomial regression analysis showing factors associated with increased duration of care home closure, November 2012–December 2016

Variable		*Univariable*				*Multivariable*			
		IRR	95% confidence interval		*P*	IRR	95% confidence interval		*P*
			Lower	Upper			Lower	Upper	
Total size of home (quartile)	1st (smallest)		*ref*				*ref*		
	2nd	1.189	1.075	1.315	< 0.001	1.154	1.038	1.283	0.008
	3rd	1.339	1.213	1.479	< 0.001	1.282	1.150	1.430	< 0.001
	4th (largest)	1.374	1.244	1.517	< 0.001	1.426	1.275	1.595	< 0.001
Total attack rate	Under 20%		*ref*				*ref*		
	20 to 39.9%	1.464	1.366	1.570	< 0.001	1.391	1.290	1.500	< 0.001
	40% and over	1.370	1.213	1.547	< 0.001	1.434	1.257	1.636	< 0.001
Winter outbreak		1.032	0.965	1.104	0.362	1.008	0.940	1.080	0.822
CQC overall rating		1.053	0.991	1.119	0.092	1.031	0.964	1.102	0.376
Residents with dementia		1.106	1.033	1.184	0.004	1.050	0.974	1.133	0.200
Ratio of residents to staff		0.914	0.846	0.986	0.023	0.977	0.895	1.068	0.613
Closure within 3 days		0.371	0.311	0.441	< 0.001	0.442	0.366	0.534	< 0.001

When adjusted simultaneously for other variables, the variable most strongly associated with decreased overall duration of closure was closure within 3 days of the first case (IRR = 0.442, 95%CI = 0.366–0.534). When adjusted for other variables, the presence of residents with dementia (IRR = 1.050, 95% CI = 0.974–1.133) and the ratio of residents to staff (IRR = 0.977, 95% CI = 0.895–1.068) were no longer significantly associated with increased duration of closure. The total size of the home remained associated with increased duration of closure (IRR = 1.426, 95% CI = 1.275–1.595 in the largest quartile), as did the total attack rate (IRR = 1.434, 95% CI = 1.257–1.595 for outbreaks with an attack rate over 40%). There was little evidence that outbreaks occurring in winter (IRR = 1.008, 95% CI = 0.940–1.080) or that the overall CQC rating of a home (IRR = 1.031, 95% CI = 0.964–1.102) were significantly associated with duration of closure.

## Discussion

In the surveillance system 795 outbreaks from 379 care homes were recorded. The attack rate of 37.1 outbreaks per 100 care homes per year is substantially higher than that observed in France (4.6 to 5.5 outbreaks per 100 facilities per year) [9] and Australia (16.8 outbreaks per 100 facilities per year) [12]. The difference in reported outbreak rates may be due to different resident populations, different structural or organisational arrangements, or lower levels of circulating pathogens at the time of surveillance. This finding could, however, represent more complete ascertainment by community health staff in frequent contact with local care homes. Ascertainment could have also been improved by the use of an internet-based reporting system, the use of which has been shown to increase the level of reporting of hospital-based norovirus outbreaks [19].

We found that care homes that closed promptly had outbreaks of shorter duration. This supports similar findings in care homes in other European countries [9, 20] and is consistent with comparable work looking at norovirus outbreaks in hospital which also found that prompt closure led to a shorter duration of outbreaks [21, 22]. We based these findings on the date of closure and the onset date of the first case. The date on which the outbreak was identified was not collected in this surveillance system, and thus we used time to closure after the first case as a proxy for outbreak identification.

We also found that increased duration of closure was significantly associated with increased size of the home and increased attack rate; both findings are epidemiologically plausible and have been observed in other studies [23]. The lack of significant association with CQC rating could be related to the scoring criteria used in this metric. Only a very minor part of the CQC rating covers topics such as infection prevention policies which have been shown to be important in preventing transmission in this setting [24]. This lack of significant association with CQC rating could also reflect the timing of the rating information; CQC data was extracted in December 2016 and therefore the ratings of a care home included in this analysis may not correspond to the rating of the care home at the point when the outbreak occurred.

Duration of closure is an important outcome as it may have a direct impact on a care home in terms of delayed admissions, and a wider impact on hospitals that are prevented from discharging patients to the affected care home. Duration of closure could be influenced by many factors. These may be organizational issues within the care home such as the time required to complete cleaning prior to re-opening and occupancy levels. As we did not capture resident capacity and therefore occupation rate, we were not able to adjust for this in our analysis. Evidence has shown that infection control measures are most effective when implemented in care homes within three days [20]. It is possible that good infection control measures slow down transmission but do not stop it, prolonging the outbreak and duration. However, information on the timing of infection control implementation, infection control policies, leadership or decontamination resources was not collected, so it was not possible to examine this. Some care homes may have taken longer to reopen as they did not have sufficient staff to undertake a deep clean promptly. This is less plausible, as the ratio of residents to staff was adjusted for in the analysis, and the significant relationship with other variables remained. In addition, it is possible that outbreaks with a high attack rate initially were more likely to be reported as they could be easier to recognize. It is plausible that the duration of these outbreaks may have been shorter due to the early onset of most cases, though it was not possible to test this, as onset dates for individual cases were not collected.

Over the four year surveillance period there were 11,568 cases, 75 hospitalisations and 29 deaths in this population; if this were extrapolated over the whole of England, this would represent a substantial burden of illness across the country. Although not directly comparable, this rate of hospitalization and mortality appears to be lower than that observed in similar settings [6]; the difference could be due to underreporting, a different population or different treatment practices. Unfortunately it was not possible to calculate incidence or morbidity measures per bed-day with the information collected in this system. Such information would be useful in order to model individual risks to residents.

We saw marked seasonality in the outbreaks, with more outbreaks occurring during the winter months (November to March). However, outbreaks were reported year round, highlighting the continuing need for good infection prevention and control practice. The winter increase we observed is in line with individual case data in hospitals [25] and the general population [26]. This seasonality in care home outbreaks may reflect the increased levels of infection circulating in the community, which increases the risk that staff, visitors or admitted residents will introduce the infection into the home. Introductions of norovirus into care homes by people are far more frequent than through food [23].

The most commonly detected pathogen was norovirus, which is consistent with studies in similar settings in other countries [9, 12]. Other viral pathogens such as rotavirus, sapovirus and astrovirus were less commonly detected. These viruses are less frequently detected in the general UK population [27] but have previously been associated with gastroenteritis outbreaks in care homes [28–30]. One of the key limitations when interpreting these findings is the large proportion (85%) of outbreaks in which samples were taken but no result was recorded. This may have been due to samples not being sent to the laboratory, not being tested at the laboratory due to procedural issues, or not being tested for viruses. Another explanation is that the database was frequently not updated with positive results from laboratory testing due to these results being reported after the surveillance report was completed. Unfortunately it was not feasible with the information stored in the surveillance database to cross-reference these results with laboratories in the area.

The primary aim of the surveillance system was to capture outbreaks of viral gastroenteritis; outbreaks of bacterial aetiology or food poisoning should have been captured on a separate incident management system and therefore not be included in this system. However, due to the syndromic nature of the case and outbreak definitions used in this surveillance system, and the small proportion of outbreaks where a sample was taken and the result recorded, some such outbreaks may have been included in this system.

One of the strengths of this analysis is that the dataset covers a large population and a wide geographical area including urban, rural and urban/rural mixed areas. It also covers a 4 year period thereby avoiding periods of unusually high or low rates of illness. One of the main limitations of this work is the difficulty of formally ascertaining the completeness of these surveillance data, both over the study period and in the different geographical areas. It might have been that ascertainment improved in the winter, leading to the observed winter increases in recorded outbreaks. Due to the close collaboration between CMHPT and CIPCTs, the data completeness is perceived to be good. Without an external dataset to validate these findings it is difficult to formally assess the completeness of these data. Nevertheless, our findings from this surveillance system are broadly consistent with other studies. Another limitation of these data is that by the nature of the surveillance system, they only include cases which are part of outbreaks. Without collecting similar information on sporadic cases of gastroenteritis, it is impossible to estimate the full burden and cause of gastroenteritis in care homes.

## Conclusions

In this study we present detailed gastroenteritis outbreak surveillance data from care homes in one area of England. This information is key to our understanding of the magnitude, cause and transmission dynamics of gastrointestinal illness in this vulnerable population. Further research is needed to understand the dynamics of and the pathogens causing gastroenteritis outbreaks in care homes, and the total associated burden of outbreak and non-outbreak infectious gastroenteritis in this population. The main finding is that closure within three days of the first case reduced significantly the duration of care home closure. This is important and has implications for care home providers and those advising on infection control practice.

## Abbreviations

CI: Confidence Interval
CIPCT: Community Infection Prevention & Control Team
CMHPT: Cheshire and Merseyside Health Protection Team
CQC: Care Quality Commission
EIA: Enzyme-linked Immunosorbent Assay
IRR: Incidence Rate Ratio
PCR: Polymerase Chain Reaction
PHE: Public Health England
